# Roles of Relative Humidity in Aerosol Pollution Aggravation over Central China during Wintertime

**DOI:** 10.3390/ijerph16224422

**Published:** 2019-11-12

**Authors:** Lin Zang, Zemin Wang, Bo Zhu, Yu Zhang

**Affiliations:** 1 Chinese Antarctic Centre of Surveying and Mapping, Wuhan University, Wuhan 430079, China; zanglin2018@whu.edu.cn; 2 Hubei Environmental Monitoring Centre, Wuhan 430079, China; zhubo125@whu.edu.cn; 3 School of Remote Sensing and Information Engineering, Wuhan University, Wuhan 430079, China; zhangyu543@whu.edu.cn

**Keywords:** aerosol pollution, hygroscopicity, relative humidity, secondary formation

## Abstract

Aerosol pollution elicits considerable public concern due to the adverse influence on air quality, climate change, and human health. Outside of emissions, haze formation is closely related to meteorological conditions, especially relative humidity (*RH*). Partly due to insufficient investigations on the aerosol hygroscopicity, the accuracy of pollution prediction in Central China is limited. In this study, taking Wuhan as a sample city, we investigated the response of aerosol pollution to *RH* during wintertime based on in-situ measurements. The results show that, aerosol pollution in Wuhan is dominated by PM_2.5_ (aerodynamic particle size not larger than 2.5 μm) on wet days (*RH* ≥ 60%), with the averaged mass fraction of 0.62 for PM_10_. Based on the *RH* dependence of aerosol light scattering (f (*RH*)), aerosol hygroscopicity was evaluated and shows the high dependence on the particle size distribution and chemical compositions. *f* (*RH* = 80%) in Wuhan was 2.18 (±0.73), which is comparable to that measured in the Pearl River Delta and Yangtze River Delta regions for urban aerosols, and generally greater than values in Beijing. Ammonium (NH_4_^+^), sulfate (SO_4_^2−^), and nitrate (NO_3_^−^) were enhanced by approximately 2.5-, 2-, and 1.5-fold respectively under wet conditions, and the ammonia-rich conditions in wintertime efficiently promoted the formation of SO_4_^2−^ and NO_3_^−^, especially at high *RH*. These secondary ions play an important role in aggravating the pollution level and aerosol light scattering. This study has important implications for understanding the roles of *RH* in aerosol pollution aggravation over Central China, and the fitted equation between *f* (*RH*) and *RH* may be helpful for pollution forecasting in this region.

## 1. Introduction

Atmospheric aerosols, consisting of large amounts of solid and liquid particles, could directly or indirectly affect the climate system [1,2,3,4]. The Intergovernmental Panel on Climate Change (IPCC) reports that aerosols are consistently the leading contributors to the uncertainty of global climate change [5,6]. What is more, numerous epidemiological studies have indicated that the suspended particles in the atmosphere, especially those with an aerodynamic diameter not larger than 2.5 µm (PM_2.5_), would produce serious adverse effects on human bodies, hence increasing deaths from cardiovascular and respiratory diseases and even lung cancer [7,8]. As such, aerosols have drawn considerable concern [9,10,11]. In recent years, numerous studies have been conducted to investigate the formation mechanisms of aerosol pollution, and some of them implied the important role of relative humidity (*RH*) in this issue because of the aerosol hygroscopicity [12,13].

On the one hand, the volume of hydrophilic aerosols increases after water uptake, thereby enhancing light scattering and, consequently, affecting horizontal visibility and the earth–atmosphere radiation budget [14,15]. To assess the effect of aerosols on the radiation budget, numerous in-situ measurements of aerosol light scattering are carried out. However, these measurements are commonly performed under dry conditions through heating air mass samples. It is necessary to transform the measured dry aerosol optical properties into corresponding values at ambient *RH* [16]. In order to quantify the relationship between the two aerosol scattering properties, the scattering hygroscopic growth factor, *f* (*RH*), was established to picture the response of aerosol optical properties to the different ambient *RH* [17]. Notably, the value of *f* (*RH*) presents significant spatial variety [16]. Quantifying *f* (*RH*) is necessary to estimate the aerosol direct radiative forcing.

On the other hand, the increased conserved water could accelerate the gas–liquid–solid reactions of gaseous precursors with particles, thereby strengthening the hygroscopicity of aerosols [14,18]. Since the 1980s, China has witnessed rapid urbanization and industrialization accompanied by a continuous increase in total emissions of air pollutants and gradual deterioration of air quality [19,20,21]. Specifically, traditional coal–smoke air pollution, which includes sulfur dioxide (SO_2_) and particles as the main components, remains severe due to the coal-dominated energy structure of China [22]. Furthermore, the concentration of photochemical precursors in the atmosphere, such as NO_x_ and volatile organic compounds, has continued to increase with vehicle ownership [23]. Other than primary emissions, secondary particle generation has become another notable cause of haze formation in the country, and may even play a decisive role occasionally [13,24,25]. Understanding the aerosol hygroscopicity is vital to further explain the physicochemical processes in the atmosphere and local haze pollution.

Notably, aerosol hygroscopicity not only has significant impacts on climate and environment, but also aggravates the threats to human health by affecting the transmission and deposition of inhaled particles into the respiratory system [26,27]. Therefore, it has elicited wide research attention, especially in the Beijing–Tianjin–Hebei (BTH) region [28,29,30], the Yangtze River Delta (YRD) region [25,31,32], and the Pearl River Delta (PRD) region [33,34]. For example, Xie et al. (2017) [25] focused on a wintertime haze episode in Shanghai in 2014, and found that both hygroscopicity and effective density of the particles increased with particle size, indicating the key role of secondary particles generated from the gaseous precursors in the particle growth. However, to the best of our knowledge, systemic studies on the aerosol hygroscopicity and its impact on pollution aggravation in Central China, one of the most polluted areas in the country, are still quite limited. Herein, observations from an environmental monitoring facility in Wuhan were adopted to explore the hygroscopic growth distributions and response of aerosol pollution to *RH* variations in Central China.

## 2. Data and Methods

### 2.1. Data

Observations from an environmental monitoring super site (114.37° E, 30.53° N) of the Hubei Environmental Monitoring Centre located in Wuhan, China, were adopted. This site is surrounded by commercial blocks and dwelling quarters, and can be considered a representative urban site. Various aerosol-related observation instruments are installed at this site, including the ambient particulate monitor (Model TEOM-1405, USA), nephelometer (Model Ecotech-Aurora1000, Australia), online ion chromatographic analyzer (Model METROHM-MAGAR 1S, Holland), automatic organic carbon (OC) and elemental carbon (EC) analyzer (Model RT4, USA), various gas analyzers and meteorological monitors, etc. The datasets used here included ground-based concentration measurements of solid and gaseous pollutants, chemical compositions in particles, and aerosol light scattering. Meteorological parameters, such as *RH* (%) and T (°C), were also obtained from in-situ measurements. Only data that met the quality requirements established by the local environmental agency were adopted, and data recorded with *RH* > 95% were excluded from this study to avoid the influence of wet deposition. The sampling time range was from December 2017 to February 2018.

The mass concentrations of PM_1_, PM_2.5_, and PM_10_, that is, particles with aerodynamic diameters not larger than 1.0, 2.5 and 10 µm, were measured using the micro-oscillation balance method with a time resolution of 1 h. Mass concentrations of main water-soluble ionic components in PM_2.5_, including cations (e.g. NH_4_^+^, Na^+^, K^+^, Mg^2+^, and Ca^2+^) and anions (e.g. SO_4_^2−^, NO_3_^−^, and Cl^−^), were measured via the ion chromatography method with a sampling velocity of 1 m^3^/h. OC and EC were monitored based on the thermal–optical transmittance method with a measuring sensitivity of 0.5 μgC/m^3^. Aerosol scattering coefficients (SC) were monitored using the integral method with a minimum detection limit of 0.3 Mm^−1^ by the nephelometer. During the experiment, the *RH* of sampled aerosols was controlled by a heating process. The mass concentrations of gas pollutants, namely, SO_2_, nitrogen dioxide (NO_2_), and ozone (O_3_), were measured through fluorescence analysis, chemi-luminescence method, and ultraviolet spectrophotometry, respectively.

In order to evaluate the influence of hygroscopic growth on aerosol observations measured by satellite remote sensing, aerosol optical depth (AOD) data provided by Himawari-8 was also used in this work. Himawari-8 is a stationary orbit satellite, which was launched in October 2014 and is operated by the Japan Aerospace Exploration Agency. Until now, only two kinds of AOD products (L2 and L3) have been published, and they have the same spatial resolution of 0.05°. However, the temporal resolution of L2 products is 10 min and that of L3 products includes 1 h, 1 day, and 1 month. There are four confidence levels, namely, very good, good, marginal, and no confidence for AOD quality assurance. Herein, hourly AOD data with the highest confidence level from L3 were adopted.

### 2.2. Method

#### 2.2.1. Parameterization of Scattering Hygroscopic Growth

The *RH* dependence of aerosol light scattering is one of the physical parameters commonly applied to describe aerosol hygroscopicity, and could be characterized by the scattering hygroscopic growth factor, denoted by *f* (*RH*), which is defined as the ratio of aerosol light scattering coefficient at a given *RH* (σ*_RH_*) and under dry conditions (σ_dry_), which is usually defined as *RH* < 40% [14,17]. This factor is used to represent the overall aerosol light scattering enhancement and is determined by the particle size distribution, chemical composition, density, and refractive index [14,35]. Several models, such as the exponential [35] and binomial models [29], have been developed to describe the relationship between *f* (*RH*) and *RH*. Herein, two widely used models, namely, the two-parameter fit equation [36,37] and the kappa equation [37,38], were adopted to investigate the hygroscopic growth for light scattering in Central China:

(1) Two-parameter fitting equation

(1) $$f\left(RH\right)=a{\left(1-\frac{RH}{100}\right)}^{-b}$$

where *a* and *b* are empirical fitting parameters. The scattering growth in this equation is normalized by the parameter of *a*, and the magnitude of the hygroscopic increase in the scattering coefficient is represented by the parameter of b [37,39]. This equation is related to both particle size and chemical composition.

(2) Kappa equation

(2) $$f\left(RH\right)=1+k\frac{RH}{100-RH},$$

where *k* is a fitting parameter, related to the average water activity of aerosol components [37]. The hygroscopic growth of aerosol scattering in this equation is theoretically expressed in terms of volume growth based on the Mie equation [37,38].

Under the condition of low *RH*, the change of *f* (*RH*) is not obvious, while at high *RH*, *f* (*RH*) changes greatly with the increase of *RH*. Based on the aircraft measurements, Beyersdorf et al. (2016) [40] concluded that at low *RH*, aerosol loadings and hygroscopic growth accounted for about 88% and 10% of the extinction variability respectively, while when *RH* > 60%, 95% of the extinction diurnal variability and 62% of the spatial variability should be attributed to aerosol water uptake. Chen et al. (2014) [14] also indicates that when *RH* < 60%, the influence of aerosol water uptake on the *f* (*RH*) is indistinct. Therefore, in order to better analyze the influence of aerosol hygroscopicity on local pollution, particular attention was paid to aerosol distribution and optical properties when *RH* ≥ 60%, defined as wet conditions here.

#### 2.2.2. Evaluation of Secondary Aerosols

Here, analysis of secondary aerosol formation focused on sulfate (SO_4_^2−^), nitrate (NO_3_^−^), and ammonium salt (NH_4_^+^), generally considered as the main composition of secondary inorganic aerosols, remarkably contributing to the moisture absorption of atmospheric particles [41]. The oxidation of gaseous precursors is the main chemical pathway for secondary particle formation. Among them, the oxidation property of gaseous precursors (SO_2_ and NO_2_) can be evaluated by the oxidation ratio, calculated following Li et al. (2013) [42] and Pani et al. (2018) [43]:

(3) $$SOR=\frac{n(SO_{4}^{2-})}{n(SO_{4}^{2-})+n(SO_{2})}$$

(4) $$NOR=\frac{n({\mathrm{NO}}_{3}^{-})}{n({\mathrm{NO}}_{3}^{-})+n({\mathrm{NO}}_{2})}$$

where *SOR* and *NOR* are the sulfur oxidation ratio and nitrogen oxidation ratio respectively, indicating the conversion degree of gas-phase SO_2_ and NO_2_ to particulate sulfate (SO_4_^2−^) and nitrate (NO_3_^−^). n(SO_4_^2−^), n(SO_2_), n(NO^3−^), and n(NO_2_) are the molar concentration of each component. Previous studies have indicated that the value of SOR (NOR) is generally less than 0.10 in the primary pollutants, and a higher SOR (NOR) denotes the significant generation of sulfates (nitrates) in the atmosphere [44,45].

## 3. Results and Discussion

### 3.1. Overview

**Figure 1:**
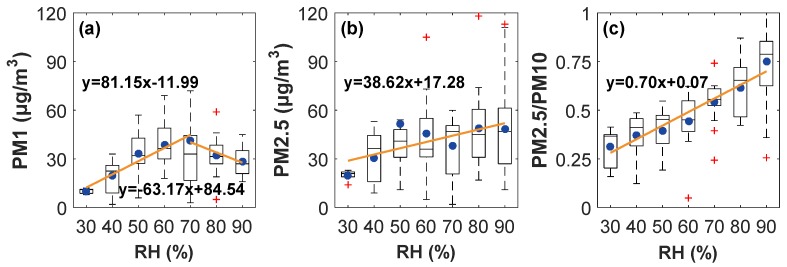
Variation in (**a**) PM_1_, (**b**) PM_2.5_ and (**c**) mass fraction of PM_2.5_ in PM_10_ with increasing *RH* during winter.

During the sampling period, the averaged concentrations of PM_1_, PM_2.5_, and PM_10_ were 29.89, 51.87, and 95.39 µg/m^3^, respectively, and the mean mass fraction of PM_2.5_ in PM_10_ was 0.55. Compared with dry conditions (*RH* ≤ 40%), although the average concentration of PM_10_ only increased by 14% on wet days (*RH* ≥ 60%), the mean contents of PM_1_ and PM_2.5_ almost doubled, which resulted in a remarkable increase in the proportion of fine particles, with the ratio of PM_2.5_/PM_10_ increasing from 0.38 to 0.62 (Table 1). When *RH* ≤ 70%, the concentrations of PM_1_ and PM_2.5_ both increased with *RH* increasing, as shown in Figure 1a,b. However, when *RH* > 70%, the concentration of PM_1_ decreased, whereas that of PM_2.5_ continued to increase. The above results suggest that the enhancement of PM_2.5_ concentration at high *RH* is dominated by particles with aerodynamic diameters between 1.0 and 2.5 µm.

### 3.2. Aerosol Scattering Hygroscopic Growth

**Figure 2:**
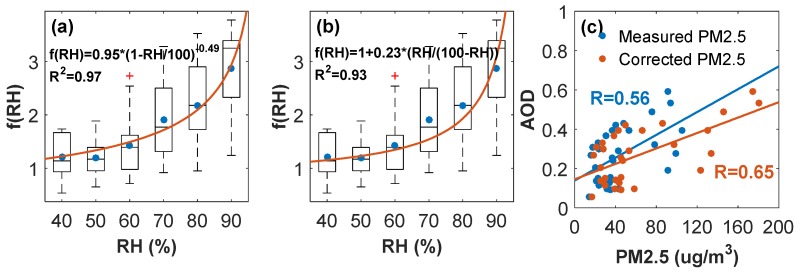
Parameterization of aerosol scattering hygroscopic growth based on (**a**) the two-parameter fitting equation and (**b**) the kappa equation. (**c**) Hygroscopic correction of satellite-based AOD during winter. Measured PM_2.5_ is dry PM_2.5_, and corrected PM_2.5_ is dry PM_2.5_ multiplied by hygroscopic growth factor.

The ambient aerosols absorb or lose water in response to the change of ambient *RH* and consequently the particle size and refractive index change, altering the aerosol scattering properties ultimately [16]. Compared with dry conditions (*RH* ≤ 40%), the averaged SC enhanced by 228.26 Mm^−1^ on wet days (*RH* ≥ 60%), from 189.71 Mm^−1^ to 441.99 Mm^−1^, which indicates the remarkable impact of aerosol hygroscopicity on the extinction for light. Here, the *RH* dependence of light scattering, *f* (*RH*), was fitted by the two-parameter fitting equation and the kappa equation respectively, as shown in Figure 2a,b. Aerosol hygroscopic growth was inconspicuous at low *RH*, only leading to slight changes in *f* (*RH*). With the increase of *RH*, especially when *RH* > 60%, aerosol hygroscopic growth leads to significant changes in light scattering.

Comparison of the fitting performance between two models indicates that the two-parameter fitting equation was more suitable for representing the actual hygroscopic growth in Wuhan in terms of a higher determination coefficient (R^2^ = 0.97). Therefore, the final parametric form of *f* (*RH*) is presented as follows:

(5) $$f\left(RH\right)=0.95*{\left(1-\frac{RH}{100}\right)}^{-0.49}$$

Based on the parametric form above, the correlation between satellite AOD from Himawari-8 and the concentration of dry particles observed from the ground stations improved from 0.56 to 0.65 after a simple hygroscopic correction considering scattering hygroscopic growth ($\mathrm{AOD}\sim {\mathrm{PM}}_{2.5}{}_{\mathrm{dry}}$ vs. $\mathrm{AOD}\sim {\mathrm{PM}}_{2.5}{}_{\mathrm{dry}}\times f\left(RH\right)$), as shown in Figure 2c.

So far, researchers in China have conducted numerous studies investigating the hygroscopic factor for aerosol scattering. In order to further analyze the difference of aerosol hygroscopicity between Central China and other regions in the country, we focused on the parameter of *f* (*RH* = 80%), which has been widely used to compare the aerosol hygroscopicity in different regions or different pollution levels, and it was summarized in Table 2. Comparisons implied that the hygroscopicity of aerosols varies in different regions with various pollution levels and aerosol types. In this study, *f* (*RH* = 80%) is 2.18 (±0.73), which is comparable to 2.4 measured in Nanjing and 2.0 (±0.3) measured in Guangzhou for urban aerosols, but it was generally greater than that measured in BTH regions. This is partly due to drier conditions and a higher dust fraction in BTH regions. Additionally, it could be found that *f* (*RH*) of urban aerosols is higher than that of rural aerosols. This may be because of the higher mass fraction of anthropogenic hydrophilic inorganic salts, organic acids, and/or organic acid salts, which need to be further investigated. The marine aerosols show the strongest hygroscopicity because of the high solubility of sea salt, which is more than twice as strong as dust aerosols.

**Figure 3:**
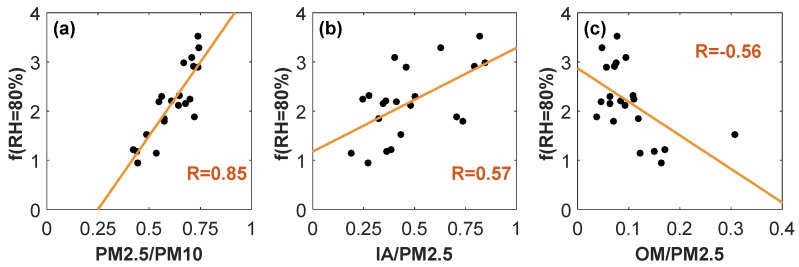
Hygroscopic growth of aerosol scattering at *RH* = 80% as a function of (**a**) mass fraction of PM_2.5_ in PM_10_, and (**b**) mass fraction of inorganic aerosols, as well as (**c**) organic matter in PM_2.5._

Herein, a sensitivity analysis of particulate scattering hygroscopic growth at *RH* = 80% was further conducted (Figure 3). There is a high linear correlation between *f* (*RH* = 80%) and the mass fraction of PM_2.5_ in PM_10_, with the correlation coefficient of 0.85, as presented in Figure 3a. With the mass fraction of inorganic salts (including NH_4_^+^, SO_4_^2−^, NO_3_^−^, Ca^2+^, Na^+^, K^+^, M^2+^, and Cl^−^) in PM_2.5_ increasing, *f* (*RH* = 80%) showed an increasing tendency, with the correlation coefficient of 0.57. Rather, a gradual decrease was observed with the enhanced mass fraction of organic matter in PM_2.5_. This is because most organic particles are hydrophobic. The similar findings were also noted by Wu et al. (2017) [29] and Chen et al. (2014) [14]. These statistical results indicate the high dependence of *f* (*RH*) on the aerosol particle size distribution and hygroscopicity, which is correlated to chemical compositions.

### 3.3. Secondary Aerosol Formation

On the basis of aerosol chemical composition measurements, Liu et al. (2014) [49] reported that three inorganic ions of NH_4_^+^, SO_4_^2−^, and NO_3_^−^, mainly generated by secondary processes, were significantly correlated to particle hygroscopic growth, while other inorganic ions (Ca^2+^, Na^+^, K^+^, M^2+^, and Cl^−^) showed little correlation. Therefore, we focus on the variation of NH_4_^+^, SO_4_^2−^, and NO_3_^−^ to further investigate the relationship between aerosol chemical compositions and *RH* in Wuhan.

**Figure 4:**
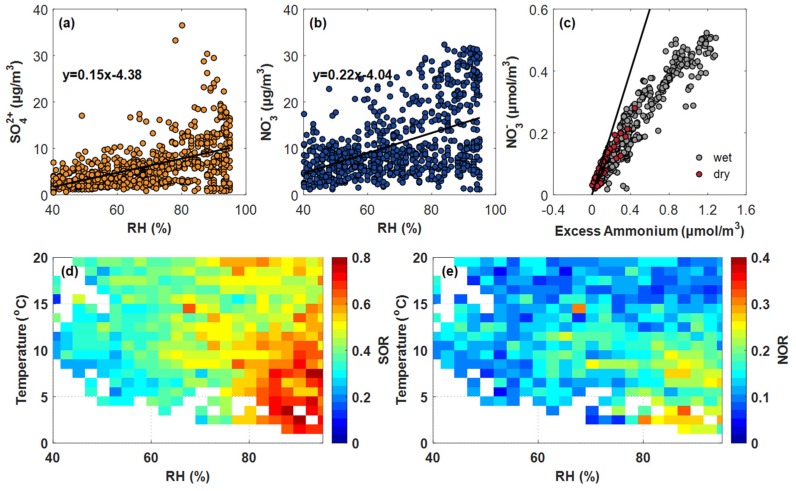
Variation of secondary inorganic salts: (**a**) SO_4_^2−^ vs. *RH*, (**b**) NO_3_^−^ vs. *RH*, (**c**) NO_3_^−^ vs. excess ammonium, (**d**) sulfur oxidation ratio, and (**e**) nitrogen oxidation ratio. The black line in (**c**) represents the 1:1 reference line.

During the sampling period, the averaged concentrations of NH_4_^+^, SO_4_^2−^, and NO_3_^−^ were 10.71, 7.53, and 6.01 µg/m^3^, respectively (Table 3). Compared with dry conditions (*RH* ≤ 40%), the mean concentrations of NH_4_^+^, SO_4_^2−^, and NO_3_^−^ increased by about 2.5-, 2-, and 1.5-fold on wet days, respectively. Figure 4 shows the detailed variation of SO_4_^2−^ and NO_3_^−^ with the increase of *RH* during winter, further suggesting *RH* plays an important role in the transformation and evolution of secondary sulfate and nitrate. SOR (Figure 4d) and NOR (Figure 4e) indicate strong oxidation during humid conditions in Wuhan, especially when *RH* > 80% and T < 10 °C.

The averaged molar ratio of NH_4_^+^ to SO_4_^2−^ was 6.7 during the sampling period, implying that Wuhan exhibits ammonia-rich conditions in wintertime. That is, SO_4_^2−^ and NO_3_^−^ could be neutralized by NH_4_^+^, and particulate sulfate and nitrate could be generated by gas-phase reactions of acid precursors with NH_3_ [50]. In accordance with Pathak et al. (2009) [51], herein we applied the excess ammonium ($\mathrm{excess}\;\left[{\mathrm{NH}}_{4}^{+}\right]=\left(\frac{\left[{\mathrm{NH}}_{4}^{+}\right]}{\left[{\mathrm{SO}}_{4}^{2-}\right]}-1.5\right)\times \left[{\mathrm{SO}}_{4}^{2-}\right]$) as a function to show the reaction between ammonia and nitric acid, and the other formation processes of nitrate in different (relative) concentrations of sulfate (Figure 4c). The result shows that the nitrate concentration increased with an almost similar increase in excess ammonium, suggesting the neutralizing process related to NH_4_^+^ plays an important role in the formation of SO_4_^2−^ and NO_3_^−^. 

**Figure 5:**
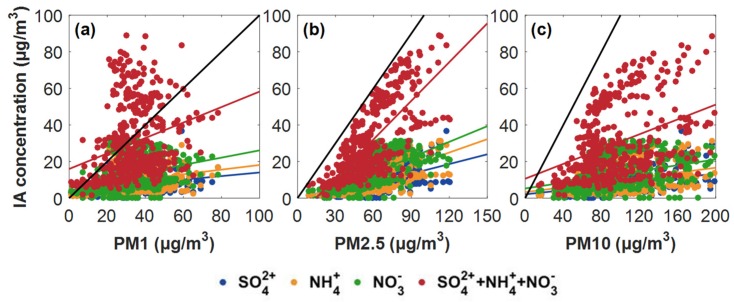
Correlation between secondary aerosol ions with (**a**) PM_1_, (**b**) PM_2.5_, and (**c**) PM_10_ under wet conditions. The black lines represent the 1:1 reference lines.

The correlation between secondary aerosol ions and particles of different sizes (i.e., PM_1_, PM_2.5_, and PM_10_) under wet conditions (*RH* > 60%) was assessed, as presented in Figure 5. The results show that secondary aerosol ions (i.e. the sum of NH_4_^+^, SO_4_^2−^, and NO_3_^−^) varied more consistently with PM_2.5_ (*R* = 0.72) than with PM_1_ (*R* = 0.24), or PM_10_ (*R* = 0.44). This phenomenon implies that secondary aerosols are mainly enriched in PM_2.5_, especially in particles with aerodynamic diameters between 1.0 and 2.5 µm, which could partly explain why PM_2.5_ and PM_1_ presented different trends with *RH* in Figure 1. These secondary ions would further promote the pollution level and light scattering.

## 4. Conclusions

Quantification of aerosol scattering hygroscopic growth is critical for determining the response of aerosol optical properties on various ambient *RH* and then modeling the aerosol direct radiative effects. In this study, in-situ measurements in Wuhan were adopted to explore the hygroscopic growth distributions and response of aerosol pollution to *RH* variations in Central China during wintertime from December 2017 to February 2018, and the main findings are as follows:

(1) Aerosol pollution in Wuhan is dominated by fine particles at high *RH*. Compared with dry conditions (*RH* ≤ 40%), the mean contents of PM_1_ and PM_2.5_ on wet days (*RH* ≥ 60%) almost doubled, and the averaged mass fraction of PM_2.5_ in PM_10_ increased to 62% from 38%. The ratio of PM_2.5_/PM_10_ presented significant increase with *RH*, however, compared with conditions of *RH* ≤ 70%, the enhancement of PM_2.5_ concentrations when *RH* > 70% should be mainly attributed to the particles with aerodynamic diameters between 1.0 and 2.5 µm, rather than PM_1_;

(2) Aerosol scattering hygroscopic growth could be well fitted by the two-parameter equation, and the correlation between satellite-based AOD and in-situ particle measurements increased from 0.56 to 0.65 after a simple hygroscopic correction based on the fitting equation, further indicating the influence of particle water uptake in the light extinction. *f* (*RH* = 80%) in Wuhan was 2.18 (±0.73), comparable to that measured in the Pearl River Delta and Yangtze River Delta regions for urban aerosols;

(3) The atmosphere showed a high oxidation property under wet conditions, especially when *RH* > 80% and T < 10 °C. Compared with dry conditions, the averaged concentrations of NH_4_^+^, SO_4_^2−^, and NO_3_^−^ on wet days increased by approximately 2.5-, 2-, and 1.5-fold, respectively, which were mainly enriched in PM_2.5_. The ammonia-rich conditions in wintertime played an important role in the formations of SO_4_^2−^ and NO_3_^−^.

This study has important implications for understanding the roles of relative humidity in aggravating aerosol pollution over Central China, and the fitted equation between *f* (*RH*) and *RH* may be helpful for pollution forecasting and evaluating aerosol radiative forcing in this region. The parameter *f* (*RH*) presents the high dependence on the aerosol particle size distribution and chemical compositions. Therefore, the new parameterization scheme for *f* (*RH*) in terms of the size distribution and chemical compositions should be developed in the future.

## Figures and Tables

**Table 1:** Variation statistics of particles under dry and wet conditions.

Items	PM_1_ (µg/m^3^)	PM_2.5_ (µg/m^3^)	PM_10_ (µg/m^3^)	PM_1_/PM_2.5_	PM_2.5_/PM_10_
Average	29.89 (13.78)	51.87 (24.03)	95.39 (37.65)	0.59 (0.19)	0.55 (0.16)
Dry (*RH* ≤ 40%)	16.11 (9.57)	31.44 (13.36)	84.83 (29.59)	0.51 (0.19)	0.38 (0.10)
Wet (*RH* ≥ 60%)	33.56 (13.04)	57.25 (20.22)	96.40 (38.19)	0.60 (0.17)	0.62 (0.14)
Increase	17.45	25.81	11.57	0.09	0.24

Note: Numbers in parentheses represent the standard deviation.

**Table 2:** Summary of scattering hygroscopic growth factors at *RH* = 80% in different regions.

Study Area	Site	Study Period	Aerosol Type	*f* (*RH* = 80%)	Reference
YRD	Nanjing	2012.11	Urban	2.4	Cui et al. (2016) [46] Cui et al. (2016) [44]
YRD	Shanghai	2011.5–2012.4	Mixed	3.5	Cheng et al. (2013) [47]
PRD	Guangzhou	2006.7	Urban Mixed Marine	2.0 (±0.3) 2.3 (±0.3) 2.7 (±0.6)	Liu et al. (2012) [33]
BTH	Rural site of Beijing	2006.4–2006.5	Dust Clean Urban	1.20 (±0.02) 1.31 (±0.03) 1.57 (±0.02)	Pan et al. (2009) [48]
BTH	Urban site of Beijing	2007.10–2007.11	Urban	1.9 (±0.3)	Liu et al. (2013) [28]
BTH	Wuqing	2009.10–2010.1	Clean Polluted	1.46 (±0.15) 1.58 (±0.19)	Chen et al. (2014) [14]
BTH	Raoyang	2014.6–2014.8	Polluted	2.28 (±0.69)	Wu et al. (2017) [29]
Central China	Wuhan	2017.12–2018.2	Urban	2.18 (±0.73)	This study

**Table 3:** Variation statistics of secondary inorganic salts and gaseous precursors during winter (µg/m^3^).

Item	NO_3_^−^	NH_4_^+^	SO_4_^2^^−^	SO_2_	NO_2_
Average	10.71 (7.56)	7.53 (6.40)	6.01 (4.82)	14.47 (11.85)	60.11 (25.99)
Dry (*RH* ≤ 40%)	5.55 (3.03)	2.68 (1.96)	2.43 (1.21)	17.54 (8.94)	51.10 (25.69)
Wet (*RH* ≥ 60%)	12.91 (8.32)	9.67 (6.97)	7.50 (5.32)	12.41 (12.63)	61.72 (25.75)
Increase	7.36	6.99	5.07	−5.13	10.62

Note: Numbers in parentheses represent the standard deviation.
